# A Genomic Approach for the Identification and Classification of Genes Involved in Cell Wall Formation and its Regulation in *Saccharomyces Cerevisiae*

**DOI:** 10.1002/cfg.85

**Published:** 2001-06

**Authors:** Piet W. J. de Groot, Cristina Ruiz, Carlos R. Vázquez de Aldana, Encarnación Dueňas, Víctor J. Cid, Francisco Del Rey, José M. Rodríquez-Peña, Pilar Pérez, Annemiek Andel, Julio Caubín, Javier Arroyo, Juan C. García, Concha Gil, María Molina, Luis J. García, César Nombela, Frans M. Klis

**Affiliations:** 1 Swammerdam Institute for Life Sciences University of Amsterdam Nieuwe Achtergracht 166 Amsterdam WV 1018 The Netherlands; 2 Departamento de Microbiologia II Facultad de Farmacia Universidad Complutense Ciudad Universitaria Madrid E-28040 Spain; 3 Instituto de Microbiología Bioquímica Departamento de Microbiología y Genética CSIC/Universidad de Salamanca Campus Miguel de Unamuno Salamanca E-37007 Spain

## Abstract

Using a hierarchical approach, 620 non-essential single-gene yeast deletants generated by EUROFAN I were systematically screened for cell-wall-related phenotypes. By analyzing for altered sensitivity to the presence of Calcofluor white or SDS in the growth medium, altered sensitivity to sonication, or abnormal morphology, 145 (23%) mutants showing at least one cell wall-related phenotype were selected. These were screened further to identify genes potentially involved in either the biosynthesis, remodeling or coupling of cell wall macromolecules or genes involved in the overall regulation of cell wall construction and to eliminate those genes with a more general, pleiotropic effect. Ninety percent of the mutants selected from the primary tests showed additional cell wall-related phenotypes. When extrapolated to the entire yeast genome, these data indicate that over 1200 genes may directly or indirectly affect cell wall formation and its regulation. Twenty-one mutants with altered levels of *β*1,3-glucan synthase activity and five Calcofluor white-resistant mutants with altered levels of chitin synthase activities were found, indicating that the corresponding genes affect *β*1,3-glucan or chitin synthesis. By selecting for increased levels of specific cell wall components in the growth medium, we identified 13 genes that are possibly implicated in different steps of cell wall assembly. Furthermore, 14 mutants showed a constitutive activation of the cell wall integrity pathway, suggesting that they participate in the modulation of the pathway either directly acting as signaling components or by triggering the Slt2-dependent compensatory mechanism. In conclusion, our screening approach represents a comprehensive functional analysis on a genomic scale of gene products involved in various aspects of fungal cell wall formation.


**Comparative and Functional Genomics**
*Comp Funct Genom* 2001; **2**: 124–142.
**DOI:** 10.1002/cfg.85

**Research Article**

### A genomic approach for the identification and classification of genes involved in cell wall formation and its regulation in *Saccharomyces cerevisiae*

Piet W. J. de Groot[1], Cristina Ruiz[2], Carlos R. Vázquez de Aldana[3], Encarnación Dueñas[3], Víctor J. Cid[2], Francisco Del Rey[3], José M. Rodríquez-Peña[2], Pilar Pérez[3], Annemiek Andel[1], Julio Caubín[2], Javier Arroyo[2], Juan C. García[3], Concha Gil[2], María Molina[2], Luis J. García[3], César Nombela[2] and Frans M. Klis[1]*

[1] *Swammerdam Institute for Life Sciences, University of Amsterdam, Nieuwe Achtergracht 166, 1018 WV Amsterdam, The Netherlands*
[2] *Departamento de Microbiologia II, Facultad de Farmacia, Universidad Complutense, Ciudad Universitaria, E-28040 Madrid, Spain*
[3] *Instituto de Microbiología Bioquímica, Departamento de Microbiología y Genética, CSIC/Universidad de Salamanca, Campus Miguel de Unamuno, E-37007 Salamanca, Spain*

**Correspondence to: Frans M. Klis, Swammerdam Institute for Life Sciences, University of Amsterdam, Nieuwe Achtergracht 166, 1018 WV Amsterdam, The Netherlands. E-mail: klis@science.uva.nl*

Received: 16 January 2001
Revised: 5 April 2001
Accepted: 9 April 2001

### Abstract

**Using a hierarchical approach, 620 non-essential single-gene yeast deletants generated by EUROFAN I were systematically screened for cell-wall-related phenotypes. By analyzing for altered sensitivity to the presence of Calcofluor white or SDS in the growth medium, altered sensitivity to sonication, or abnormal morphology, 145 (23%) mutants showing at least one cell wall-related phenotype were selected. These were screened further to identify genes potentially involved in either the biosynthesis, remodeling or coupling of cell wall macromolecules or genes involved in the overall regulation of cell wall construction and to eliminate those genes with a more general, pleiotropic effect. Ninety percent of the mutants selected from the primary tests showed additional cell wall-related phenotypes. When extrapolated to the entire yeast genome, these data indicate that over 1200 genes may directly or indirectly affect cell wall formation and its regulation. Twenty-one mutants with altered levels of $\beta$1,3-glucan synthase activity and five Calcofluor white-resistant mutants with altered levels of chitin synthase activities were found, indicating that the corresponding genes affect $\beta$1,3-glucan or chitin synthesis. By selecting for increased levels of specific cell wall components in the growth medium, we identified 13 genes that are possibly implicated in different steps of cell wall assembly. Furthermore, 14 mutants showed a constitutive activation of the cell wall integrity pathway, suggesting that they participate in the modulation of the pathway either directly acting as signaling components or by triggering the Slt2-dependent compensatory mechanism. In conclusion, our screening approach represents a comprehensive functional analysis on a genomic scale of gene products involved in various aspects of fungal cell wall formation. Copyright © 2001 John Wiley & Sons, Ltd.**

**Keywords:** **functional genomics; $\beta$-glucan; mannoproteins; chitin; morphogenesis**

### Introduction

The cell wall of *S. cerevisiae* is an essential organelle whose rigid structure determines cell shape, enables cells to withstand internal turgor pressure and protects cells against environmental stresses. The four major components of the cell wall are the polysaccharides $\beta$1,3-glucan, $\beta$1,6-glucan and chitin, and various mannoproteins. These may be covalently linked to each other to form macromolecular complexes (Kollár *et al.*, 1995, 1997; Kapteyn *et al.*, 1999; Smits *et al.*, 1999). Two types of cell wall

Copyright © 2001 John Wiley & Sons, Ltd.

proteins (CWPs) covalently linked to glucans, namely GPI-CWPs (containing a glycosylphosphatidylinositol-derived structure) and Pir-CWPs (for Proteins with Internal Repeats), have been identified. The composition and structure of the yeast cell wall may vary considerably both during cell cycle progression (Klis, 1994; Cid *et al.*, 1995), or when exposed to changing environmental conditions, e.g. heat stress (Jung and Levin, 1999) or the presence of pheromones (Klis, 1994; Cid *et al.*, 1995). When yeast cells are grown in the presence of cell wall or cell membrane perturbing agents (Calcofluor white, SDS), cell wall construction is also adapted (Ketela *et al.*, 1999). Studies with mutants lacking *FKS1* or *GAS1*, involved in synthesis (Mazur *et al.*, 1995) and remodeling of $\beta$1,3-glucan chains (Popolo and Vai, 1999; Mouyna *et al.*, 2000), respectively, showed that in these mutants a compensatory mechanism is induced (Popolo *et al.*, 1997; Dallies *et al.*, 1998; Ram *et al.*, 1998). As a result, chitin levels in the cell wall rise and more cell wall mannoproteins become bound, through $\beta$1,6-glucan, to chitin (Kapteyn *et al.*, 1997). In addition, the cell walls contain more Cwp1 and more Pir proteins, and transcription of *FKS2*, which encodes a catalytic subunit of the $\beta$1,3-glucan synthase complex, is upregulated (Kapteyn *et al.*, 1999; Ram *et al.*, 1998).

Partly due to its potential as a selective target for antifungal drugs, a great effort has been made in recent years to characterize genes involved in the construction and maintenance of the yeast cell wall. This has resulted in the cloning and characterization of genes involved in different aspects of cell wall biosynthesis, such as $\beta$1,3-glucan synthesis (*FKS1*, *FKS2*, Mazur *et al.*, 1995), processing of $\beta$1,3-glucan chains (Popolo and Vai, 1999; Rodriguez-Peña *et al.*, 2000), $\beta$1,6-glucan synthesis (Shahinian and Bussey, 2000) and chitin synthesis (*CHS1* to *CHS6*, reviewed in Bulawa, 1993, and *CHS7*, Trilla *et al.*, 1999). However, much less is known about genes that encode proteins involved in coupling reactions between cell wall macromolecules. In addition to genes directly involved in cell wall construction, a number of genes that regulate biosynthesis of the cell wall through modulation of the protein kinase C-directed cell wall integrity pathway have been characterized. This pathway, essential for maintaining a stable cell wall, is activated in response to a variety of stress conditions that trigger a compensatory mechanism (Martín *et al.*, 2000; De Nobel *et al.*, 2000). It has recently been shown that most of the genes controlled by this pathway encode known or putative cell wall proteins and enzymes involved in cell wall biogenesis (Jung and Levin, 1999).

A genomic approach to further extend our knowledge of the cell biology of *S. cerevisiae* was undertaken by a EUROpean Functional Analysis Network for the yeast genome (EUROFAN). In this program, strains deleted in genes of unknown function generated by EUROFAN I were systematically screened using hierarchical approaches (EUROFAN II). As members of EUROFAN, we have analyzed the collection of deletants to improve our understanding of cell wall biogenesis of *S. cerevisiae*. For this purpose, in a first round of screens, we selected for mutants that appear to have altered cell walls by analyzing the sensitivity of cells challenged with the cell wall and cell membrane perturbing compounds Calcofluor white and SDS, and a sonication procedure. Additionally, cells were studied by light microscopy for having morphological defects. The mutants thus selected were further analyzed for additional cell wall phenotypes in a hierarchical manner using screens with increasing specificity and discriminatory power. These screens enabled us to identify candidate genes coding for structural cell wall proteins, proteins involved either in the biosynthesis, processing or assembly of the different cell wall macromolecules, and those involved in the regulation of cell wall formation. At the same time, these screens helped us to validate the assays chosen for the primary selection of potential cell wall-related mutants. Our hierarchical large-scale screening approach therefore allows the efficient identification of genes involved in different aspects of cell wall formation and its regulation. We propose that a similar approach will be effective for other fungi as well.

### Materials and methods

### Strains and media

All EUROFAN deletants used in this study are haploid derivatives of the EUROFAN reference strain FY1679 (*MAT***a**/$\alpha$ *ura3-52*/*ura3*-52 *leu2*$\Delta$*1*/+ *his3*$\Delta$200/+ *trp1*$\Delta$*63*/+ *GAL2*/*GAL2*) (Winston *et al.*, 1995). *S. cerevisiae* strain FYDK (*MAT***a** *slt2*$\Delta$ *ura3*-52 *leu2*$\Delta$*1* *his3*$\Delta$200 *trp1*$\Delta$*63*) was used as a control for immunoblot analysis of Slt2

Copyright © 2001 John Wiley & Sons, Ltd. 

phosphorylation (De Nobel *et al.*, 2000). The killer toxin producing strains are *S. cerevisiae* 2629 (K1) and GY-2-3a (K28), and *Hansenula mrakii* strain IFO 0895 (HM-1).

For routine culturing, *S. cerevisiae* was grown on YED (1% (w/v) yeast extract and 2% (w/v) glucose) or YEPD (YED + 2% (w/v) peptone) medium at 28°C. For the calcium assay, YED plates were prepared with high purity agarose (Merck) instead of agar to solidify the medium, and $CaCl_2$ was added to the medium after sterilization to avoid precipitation. For immunoblot-analyses of medium components, yeast strains were grown at 30°C in synthetic complete (SC) medium (0.17% (w/v) yeast nitrogen base without amino acids and ammonium sulfate (Difco), 2% (w/v) glucose, 0.5% (w/v) casamino acids, 0.5% (w/v) ammonium sulfate, 0.25% (w/v) succinate, 0.01% (w/v) uracil, 0.01% (w/v) tryptophan, and 0.01% (w/v) adenine sulfate) at pH 5.5.

### Calcofluor White and SDS spot assays

The spot assay used to screen for hypersensitivity or resistance to Calcofluor White was adapted from the assay described by Ram *et al.* (1994). Calcofluor white plates were prepared by adding Calcofluor white (from a 1% (w/v) stock solution) to sterile YEPD medium at 70°C to a final concentration of 50 μg/ml. On these plates, 3 μl drops were spotted that contained $\sim 10^5$, $10^4$, $10^3$, $10^2$ and 10 cells/μl, respectively, and after two days at 30°C growth was scored.

Similar to the Calcofluor white assay, mutants that are sensitive to SDS were identified by adding SDS (400 μg/ml) for *TRP1* strains and (12 μg/ml) for *trp1* auxotrophs) to the YEPD medium.

### Sonication assay

The sonication protocol was adapted from Ruiz *et al.* (1999). Cells were grown overnight in 5 ml YED cultures. One ml of each cell suspension was sonicated for 30 seconds on ice at a wave amplitude of 2 microns in a Vibra Cell (Sonic & Materials, Connecticut, USA). The percentage of sonication-sensitive cells was determined by the addition of 300 μl 0.005% propidium iodide (PI) to 100 μl of untreated (control) and treated cells, and measuring the number of PI positive cells using a FACStar Plus flow cytometer (Becton Dickinson, San Jose, CA). The relative sonication-sensitivity or resistance of each mutant was determined by calculating the ratio of the percentage of damaged cells of the mutant and that of the isogenic wild type due to sonication. Mutants with a ratio $\geq 1.5$ were considered sonication-sensitive, while those with a ratio $\leq 0.5$ were defined as resistant.

### Calcofluor White staining and fluorescence microscopy

The protocol was adapted from Pringle (1991) for screening large numbers of strains. Cells from an overnight culture (28°C in YED), were inoculated 1 : 10 in fresh YED medium. To enhance the detection of cell wall-related phenotypes, the cells were incubated for 5 h at 37°C. From each culture a 200 μl sample was taken for centrifugation (1 min) and the supernatant removed. Cells were resuspended in 50 μl of a 10 μg/ml solution of Calcofluor white (Fluorescent Brightener 28, Sigma) and observed using an Olympus IMT-2 fluorescence microscope provided with an Olympus BH2-RFL-T3 lamp, an appropriate set of filters (UV to blue for excitation and blue to green for emission) and a 100 × immersion objective.

### Immunoblot analysis of Slt2 activation

Slt2 activation was determined basically as described by Martín *et al.* (2000). Briefly, cells were grown to mid-log phase, diluted to $A_{600} = 0.2$ and grown for one generation at 24°C or 39°C. After cell breakage, cell extracts were separated from glass beads and cell debris by centrifugation and collected in new tubes. Protein concentrations ($A_{280}$) were determined and 80 μg protein samples were fractionated by SDS-PAGE and analysed by Western blotting. Membranes were probed with anti-phospho-p44/42 MAPK (Thr202/Tyr204) antibodies (New England Biolabs) to detect dually phosphorylated Slt2, and then stripped and reprobed with anti-GST-Slt2 antibodies (Martín *et al.*, 1993) in order to monitor the total amount of Slt2 in each lane.

### Caffeine sensitivity and sorbitol remediation

Cells were grown overnight at 28°C in YED medium. Five μl cell suspensions containing $10^4$ cells were spotted on YED, YED + 12 mM caffeine

Copyright © 2001 John Wiley & Sons, Ltd.

(Sigma) and YED + 12 mM caffeine + 0.5 M sorbitol (Merck) plates. Growth was scored after two days at 28°C for YED plates and after three days for the caffeine-containing ones. Strains that showed sensitivity to caffeine were further studied by spotting serial tenfold dilutions ($\sim 10^4$, $10^3$, $10^2$ and 10 cells).

### Calcium chloride and lithium chloride spot assays

Cells suspensions as described above for the caffeine sensitivity assay were spotted on YED and YED + 0.2 M $CaCl_2$ plates. Growth was scored after two days at 28°C. Sensitivity to 0.2 M LiCl was evaluated by an equivalent procedure in order to discern strains that are calcium-specific from those that are sensitive to high ionic conditions. Strains that showed sensitivity to calcium and not to lithium were further studied by spotting serial tenfold dilutions ($\sim 10^4$, $10^3$, $10^2$ and 10 cells).

### Zymolyase assay

Sensitivity to Zymolyase was determined based on the method described by Lussier *et al.* (1997). Cells from strains grown overnight in YEPD were washed in water and resuspended in 10 mM Tris-HCl pH 7.4, at a concentration of $10^7$ cells/ml. Zymolyase-20T (Seikagaku Corporation, Tokyo, Japan) was added at a concentration of 5 µg/ml and the cell density at the start of the incubation was measured as $A_{600}$. Incubations were performed for 4 h at 37°C and $A_{600}$ was read at one-hour intervals to make sure that the decrease in cell density was in the linear range. Mutants were considered sensitive to Zymolyase when the $A_{600}$ after a 4 h incubation was 70% or less compared to that of the FY1679a strain and resistant when it was 130% or higher.

### Killer toxin assays

Sensitivities to killer toxins K1, K28 and HM-1 were determined using a seeded plate assay (Boone *et al.*, 1990). Killer toxin-producing strains were grown overnight on YEPD plates at 30°C and cells from these plates were resuspended in water to obtain concentrated cell suspensions. Strains to be tested were grown overnight in 5 ml YEPD, washed with sterile water and resuspended at a cell density of $A_{600} = 0.5$. Seeded plate medium consisted of 20 ml YEPD-agar supplemented with 50 mM sodium citrate buffer (pH 3.7–3.8) and 30 µg/ml methylene blue. This medium was inoculated with 100 µl cell suspension of strains to be tested and the plates were allowed to dry completely before spotting 3 µl of the killer toxin producing strains. For each deletion strain to be tested two plates were prepared, one of which was incubated at 22°C and another at 30°C for two to three days. Resistance/sensitivity was determined by comparing the growth inhibition halos of mutants with the halo of the haploid reference strain FY1679a.

### Cyclosporin sensitivity assay

Cells were grown overnight at 28°C in YED medium and 200 µl cell suspensions containing $10^3$ cells were deposited in wells of 96-well microtiter plates. For each mutant three conditions were used; YED medium, YED + 50 µg/ml Cyclosporin A (Sandoz Pharma AG, Basel) and YED + 100 µg/ml Cyclosporin A. Plates were incubated one day at 28°C and optical density was measured to determine the level of growth.

### Papulacandin B and Echinocandin B assays

Sensitivities to the glucan synthase inhibitors Papulacandin B (Ciba-Geigy, Basel, Switzerland) and Echinocandin B (Eli Lilly, Indianapolis, USA) were determined by spotting serial dilutions of cell suspensions, as described for the Calcofluor white spot-assay, on YEPD plates containing the inhibitors. The concentrations used were 1 µg/ml Papulacandin B and 0.2 µg/ml Echinocandin B, respectively.

### *In vitro* $\beta$1,3-Glucan synthase and chitin synthase activities

For determination of $\beta$1,3-glucan synthase and chitin synthase activities, cells grown to mid-logarithmic-phase were homogenized and the membrane fraction was isolated by centrifugation at 48.000 × g for 30 min. ChsI, ChsII, and ChsIII activity measurements on the membrane fractions were performed as described by Choi and Cabib (1994), and $\beta$1,3-glucan synthase activity was determined as described by Ishiguro *et al.* (1997)

Copyright © 2001 John Wiley & Sons, Ltd.

either with 150 μM GTP or without GTP in the assay mixture. Glucan and chitin synthase activities were defined as the amount of glucose and *N*-acetylglucosamine incorporated into glucan and chitin, repectively. Enzyme activities of deletion mutants per milligram of protein were compared to the wild type strain FY1679a and were considered as being increased if $>120\%$ and decreased if $<80\%$ compared to wild-type.

### Immunoblot analysis of culture supernatant

Yeast strains were grown in SC to mid-log phase ($A_{600} \sim 2$). Cells and culture media were carefully separated by centrifugation ($2 \times 10$ min at $1{,}600 \times g$). Culture supernatants of the different strains were diluted to a concentration equivalent to $A_{600} = 1.6$ with 10 mM Tris-HCl, pH 6.8, and from these solutions, two-fold serial dilutions were prepared. These dilutions were spotted onto Immobilon-NC (Millipore) using a Bio-Dot apparatus (Biorad). For Western analysis using β1,3-glucan antibodies, Immobilon-P (Millipore) instead of Immobilon-NC was preferred. Spots were allowed to dry overnight. Western immunoblot analyses were performed according to Klis *et al.* (1998) using monoclonal antibodies against β1,3-glucan (Bio-supplies Australia) and polyclonal antisera raised against β1,6-glucan-BSA (Kapteyn *et al.*, 1995), Ssr1 (Moukadiri *et al.*, 1997), Cwp1 (Shimoi *et al.*, 1995), and Pir2/Hsp150 (Russo *et al.*, 1992). Western blots were visualized with ECL Western blotting detection reagents (Amersham) according to the manufacturer's instructions. Signal intensities of spots were determined by densitometric scanning of spots in the linear range of the X-ray films. A mutant was considered to have increased levels of a certain cell wall component in the medium when the amount was $\geq 1.7\times$ the wild type level.

For SDS-PAGE analysis of medium proteins, proteins were precipitated overnight in 80% cold ethanol and washed twice with 80% acetone. Precipitated proteins corresponding to the equivalent of 200 μl culture supernatant of cells grown to $A_{600} = 0\ 2$ were separated by electrophoresis using linear 2.2–20% polyacrylamide gels and electrophoretically transferred onto Immobilon polyvinylidene (PVDF) membranes (Montijn *et al.*, 1994). Immunoblot analyses were performed as described above.

### Results

### General approach

Deletion mutants generated by EUROFAN I (Oliver, 1996) were systematically screened for cell wall-related phenotypes. Efficient analysis of the whole collection of EUROFAN I mutants was achieved by using a hierarchical screening approach (Figure 1). This method employed rapid screening of all mutants with easy-to-perform assays in order to select potential cell wall-related genes. To elucidate which aspect of cell wall formation was affected, the mutants that scored positively were analyzed further using tests of increasing specificity. The detailed results of our screens are compiled in an EUROFAN database that is accessible at: **http://www.mips.biochem.mpg.de/proj/eurofan/eurofan_2/n7/index.html**

### Primary screens for the identification of cell wall-related genes

For primary identification of potential cell wall-related genes, the 620 EUROFAN mutants, each deleted in an individual ORF, were analyzed using phenotypic assays which are indicative of mutations leading to a defective cell wall. Our primary tests scored for altered sensitivity to Calcofluor White, SDS, sonication, and for abnormal morphologies. Calcofluor White is a fluorescent agent that binds to chitin and interferes with the polymerization of this cell wall component (Elorza *et al.*, 1983; Roncero and Durán, 1985). For this reason, it has been widely used for chitin staining (Pringle *et al.*, 1989) but also for the identification of cell wall-related mutants after random mutagenesis (Roncero *et al.*, 1988; Ram *et al.*, 1994; Lussier *et al.*, 1997). SDS is a detergent that affects membrane stability and indirectly also cell wall construction. It can thus be used to reveal cell wall defects that result in increased accessibility of SDS to the plasma membrane (Shimizu *et al.*, 1994; Igual *et al.*, 1996; Bickle *et al.*, 1998). The use of sonication is also a powerful tool for the identification of cell wall alterations; in addition, a procedure applicable at large scale has been developed (Ruiz *et al.*, 1999). Finally, the cell wall is responsible for maintaining cell shape and, therefore, morphological abnormalities may be indicative of alterations in cell wall dynamics during morphogenesis. Microscopic observation of cells stained with Calcofluor white

Copyright © 2001 John Wiley & Sons, Ltd.

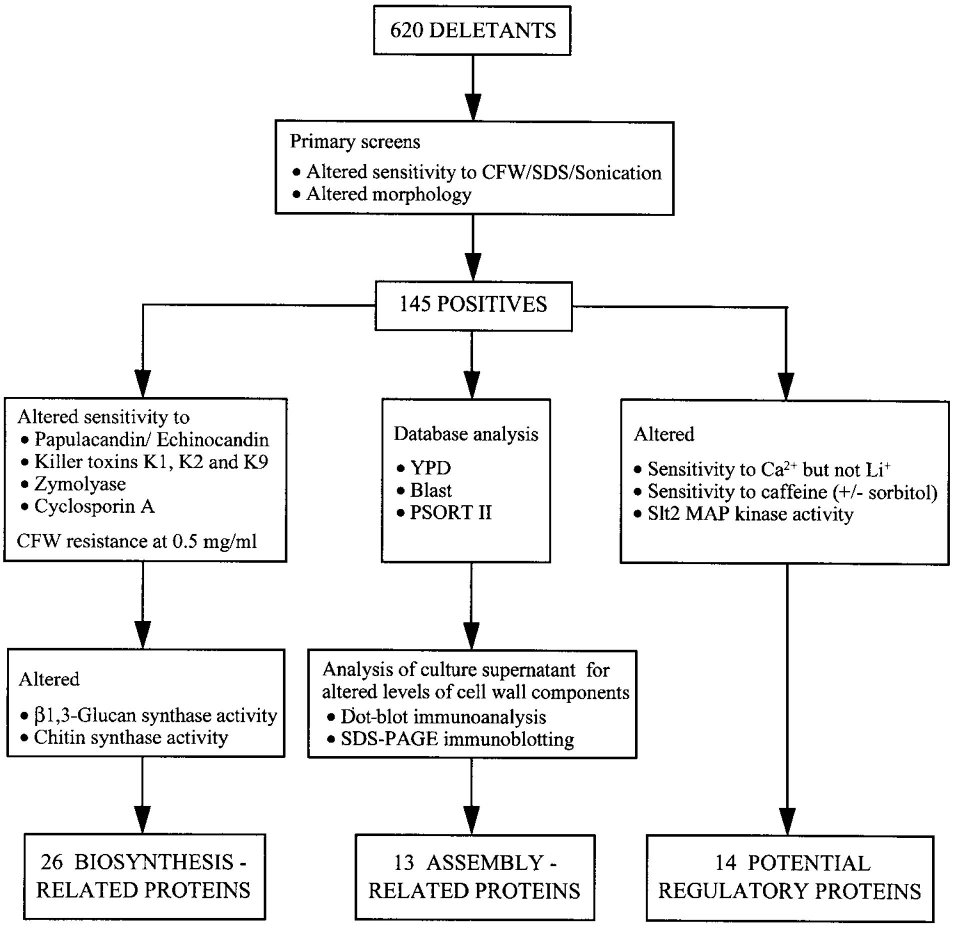

**Figure 1.** Hierarchical screening approach for the identification of proteins involved in cell wall formation and its regulation allowed the identification of mutants showing different chitin deposition patterns and other alterations related to morphogenesis, such as abnormal septation, hyperpolarized growth or abnormal budding patterns. Examples of mutants displaying a variety of morphogenetic defects are shown in Figure 2.

In total, among the 620 mutants that were analyzed, 145 (23%) mutants showed phenotypic abnormalities in at least one of the screens used (Table 1). The number of mutants selected by each primary screen ranged from 51 mutants (8.2%) showing abnormal morphology to 90 mutants (14.5%) with altered sensitivity to Calcofluor white. The sets of mutants obtained with the single screens showed limited overlap. For example, 35% and 39% of the sonication-sensitive mutants showed altered sensitivity to SDS and Calcofluor, respectively, and indeed, only six of the 145 mutants selected by the primary screens were positive in all four primary screens. This indicates that our primary screens are largely independent from each other. More extensive overlap was found only in one case: 63% of the sonication-sensitive mutants had an abnormal shape, supporting the idea that mutants affected in morphogenetic processes have defects at particular sites in the cell wall which might lead to loss of cell integrity after sonication (Cid *et al.*, 1998; Ruiz *et al.*, 1999).

### Specific screens for the identification of proteins involved in cell wall biosynthetic reactions

In a second round of screens, the 145 mutants selected by primary analysis were further analyzed

Copyright © 2001 John Wiley & Sons, Ltd.

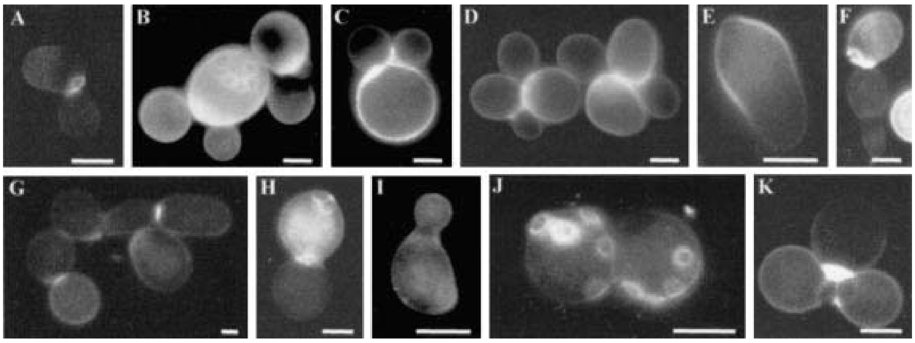

**Figure 2.** Typical morphological mutants. Cells were grown at 37°C for five hours and subsequently stained with Calcofluor white. (A) WT (FY1679, haploid segregant); (B) *fks1*Δ (deficient in β-1,3-glucan synthase activity), characteristic cell aggregate, showing enhanced fluorescence due to high chitin content; (C) *ybr078w*Δ (*ecm33*Δ), spherical, swollen and chitinous; (D) *ydl005c*Δ, aggregated cells; (E) *ydl074c*Δ (*bre1*Δ), irregularly-shaped cells; (F) *ybr133c*Δ (*hsl7*Δ), hyperpolarized cells; (G) *ylr024c*Δ, large cells; (H) *ygr262c*Δ, doublets of large round cells with asymmetrical chitin deposition; (I) *ynl233w*Δ (*bni4*Δ), cells lacking a chitin-enriched neck and, subsequently, lacking visible bud scars. (J) *yol093w*Δ, cells with altered budding pattern; (K) *ydl225w*Δ (*sep7*Δ), cells with cytokinetic defects (failure to form normal septa, leading to a variety of defects in the pattern of chitin deposition at the neck, like asymmetrically thickened septa). The bars indicate 5 μm using screens of higher specificity in order to discriminate between mutants related to different aspects of cell wall formation and mutants that present a more general phenotype (Figure 1). We have not screened for mannosylation and phosphorylation defects of cell wall proteins. For this we refer to the results of the Eurofan II Secretion and Protein Trafficking Node at MIPS **http://www.mips.biochem.mpg.de/proj/eurofan/eurofan_2/n5/index.html.**

Table 1. Identification of cell wall-related mutants by primary screens and their validation by secondary screens

| Primary screens | Positive mutants | | |
|---|---|---|---|
| Altered sensitivity to | Increased sensitivity | Decreased sensitivity | Total |
| ● Calcofluor white | 67 (61) | 23 (21) | 90 (82) |
| ● SDS | 58 (57) | nd | 58 (57) |
| ● Sonication | 46 (40) | 11 (9) | 57 (49) |
| Altered morphology | | | 51 (48) |
| Positive in one or more primary screens | | | 145 (131) |

nd, not determined.
Six hundred and twenty deletion mutants were screened. Deletants that scored positively in one or more of the primary screens (145 strains), were further analyzed (Figure 1). The number of mutants that also scored positively in one or more secondary tests are presented between parentheses. On average, 90% of the positives in the primary screens scored also positively in one or more of the secondary screens. The data for each mutant can be found at MIPS: **http://www.mips.biochem.mpg.de/proj/eurofan/eurofan_2/n7/index.html**.

To identify genes encoding proteins belonging to cell wall biosynthetic enzyme complexes or proteins involved in the modulation of the synthetic activities, we analyzed the 145 selected mutants for their sensitivities to the β1,3-glucan synthase inhibitors Papulacandin B and Echinocandin B, to the killer toxins K1, K28, and HM-1, which bind to specific cell wall components, to Zymolyase, an enzyme cocktail comprising β1,3-glucanase and protease activities, and to the calcineurin inhibitor Cyclosporin A. Papulacandin B and Echinocandin B act either by hindering some components of the β1,3-glucan synthase complex or by inhibiting the incorporation of the glucans into the extracellular matrix (Font de Mora *et al.*, 1993; Ram *et al.*, 1994). Interestingly, although resistance or sensitivity to both inhibitors are usually correlated, we found a group of deletants that are sensitive to one of these drugs and behave as wild type for the other, pointing out that the mechanism of action of both compounds is different (Table 2). Increased resistance of mutant cells to the killer toxin K1 has often been found to correlate with low levels of β1,6-glucan, the receptor molecule for this toxin, in the wall (Shahinian and Bussey, 2000). Similarly, low levels of mannan are expected to correlate with increased resistance to killer toxin K28 (Schmitt and Radler, 1987) and low levels of β1,3-glucan with increased resistance to killer toxin HM-1

Copyright © 2001 John Wiley & Sons, Ltd. 

Table 2. Mutants with altered $\beta$1,3-glucan synthase activities

| **ORF** | | | **Additional phenotypes[4]** | | | | | |
|---|---|---|---|---|---|---|---|---|
| **Decreased GS[1] activity** | **Protein name[2]** | **Putative biochemical function or cellular role[3]** | **PC** | **EC** | **K1** | **K28** | **HM-1** | **Ca** |
| *YBL024w* | Ncl1 | RNA processing/modification | S | | S | | S | S |
| *YDL005c* | Med2 | Polymerase II transcription | (S) | (S) | | S | S | S |
| *YDL074c* | Bre1 | Unknown | (S) | S | | S | S | S |
| *YDL077c* | Vam6 | Vesicular transport | S | | | S | S | S* |
| *YDL115c* | | Unknown | | S | S | S | S | S |
| *YDR071c* | | Unknown | | R | | S | S | S |
| *YJL029c* | Vps53 | Vesicular transport | | (S) | | | S | S* |
| *YJL124c* | Lsm1 | RNA turnover | | (S) | S | S | S | S |
| *YJR059w* | Ptk2 | Protein kinase | R | | S | S | S | S |
| *YNL059c* | Arp5 | Actin-related protein | S | S | S | S | S | S* |
| *YNL225c* | Cnm67 | Nuclear migration | | S | S | S | S | |
| *YOL018c* | Tlg2 | Vesicular transport | S | S | | | S | S* |
| *YOL072w* | Thp1 | Unknown | | | | S | | S* |
| *YOL124c* | | Unknown | R | | R | S | S | |
| Increased GS activity | | | | | | | | |
| *YDL146w* | | Unknown | (S) | | | | | |
| *YGL012w* | Erg4 | Fatty-acid metabolism | S | S | R | | R | |
| *YGL100w* | Seh1 | Nuclear-cytoplasmic transport | | S | R | | S | S* |
| *YGR200c* | Elp2 | Polymerase II transcription | | (S) | | | | |
| *YGR210c* | | Unknown | | | | S | S | |
| *YJL006c* | Ctk2 | Polymerase II transcription | (S) | (S) | | nd | | S* |
| *YNL106c* | Inp52 | Inositol polyphosphate phosphatase | | (S) | | | | |

[1]GS, Levels of $\beta$1,3 glucan synthase activity compared to the wild-type strain.
[2]YPD annotation.
[3]From YPD or MIPS.
[4]PC, Papulacandin B; EC, Echinocandin B; K1, K28 and HM-1, killer toxins; Ca, Calcium; S, hypersensitive; (S), slightly more sensitive than wild-type, S*, Calcium and Lithium hypersensitive; R, resistant; nd, not determined.

(Kasahara *et al.*, 1994). Altered Zymolyase sensitivity may reflect changes in the $\beta$1,3-glucan layer (Ovalle *et al.*, 1998) or changes in the external mannoprotein layer that result in altered permeability to cell wall degrading enzymes (De Nobel *et al.*, 1991). Increased sensitivity to K1, K28, HM-1 and Zymolyase was found for 29, 50, 76 and 42 mutants, respectively whereas increased resistance to K1, HM-1 and Zymolyase was found for 17, 8 and 2 mutants respectively. For the K28 toxin, we only scored for increased sensitivity because the growth inhibition halo of the wild type strain was rather small. Of the K1-hypersensitive mutants, all except one were also hypersensitive to the HM-1 toxin, indicating that the amount of $\beta$1,6-glucan that will be incorporated in the cell wall strongly depends on the amount of $\beta$1,3-glucan present in the wall. This was expected since the $\beta$1,6-glucan in the wall is covalently linked to the $\beta$1,3-glucan network. Twenty-nine mutants were hypersensitive to both Zymolyase and killer toxin HM-1, indicating that these mutants at least seem to have alterations in their $\beta$1,3-glucan layer. It is well known that Cyclosporin A functions as a calcineurin inhibitor that negatively affects *FKS2* expression (Zhao *et al.*, 1998), and sensitivity to this drug is therefore related to glucan synthesis. *FKS2* codes for one of the subunits of the glucan synthase complex whose expression is increased in the absence of Fks1 function (Mazur *et al.*, 1995; Ram *et al.*, 1998). Sensitivity of *fks1*$\Delta$ mutants to Cyclosporin A is therefore due to its effect on *FKS2* expression. We detected only one mutant, *yjl029c*$\Delta$, that was hypersensitive to this drug. This mutant also showed diminished glucan synthase activity (see below and Table 2), supporting the idea that the activity of Yjl029c is directly related to glucan biogenesis.

The tests described above indicated that among the 145 selected mutants, a group of mutants may

Copyright © 2001 John Wiley & Sons, Ltd. 

be affected in the synthesis of $\beta$1,3 glucan, $\beta$1,6 glucan or mannan. To obtain more direct evidence that some of the proteins are involved in the activity of the $\beta$1,3 glucan synthase complex, we determined the *in vitro* activity of this enzyme in 50 mutants selected on the basis of results obtained in the killer, Zymolyase or Papulacandin B/Echinocandin B assays (Table 2). 21 of these mutants have significantly altered levels of $\beta$1,3-glucan synthase activity, either lowered or increased, compared to the wild type strain, indicating that in these mutants the deletion involved a protein that is required for normal $\beta$1,3-glucan synthase activity. For each mutant the relative glucan synthase activities in the absence and presence of GTP correlated (not shown), indicating that in none of the deletants was the activation of $\beta$1,3-glucan synthase by GTP affected. 13 of the 14 mutants with reduced glucan synthase activity were hypersensitive to the HM-1 killer toxin and 11 of them also showed hypersensitivity towards K28 toxin. This suggests that the increased killer sensitivity in these mutants may be due to their lower levels of $\beta$1,3-glucan and/or probably altered levels of mannoproteins in the cell wall. In seven mutants, an increase of $\beta$1,3-glucan synthase activity was detected suggesting that perhaps proteins involved in negative regulation of $\beta$1,3-glucan synthesis are affected. 14 of the 21 mutants with altered $\beta$1,3-glucan synthase activity are deleted in currently identified genes. Among these is a group of genes that is responsible for a wide range of functions in the cell (such as *NCL1*, *MED2*, *SEH1*, *ELP2*, *CTK2* or *PTK2*) suggesting that the observed effects on $\beta$1,3-glucan synthase activity may be indirect. Others perform cellular functions that are more directly related to cell wall construction, morphogenesis or secretion (*VAM6*, *VPS53*, *ARP5*, *INP52, TLG2*), but defects in $\beta$1,3-glucan synthase have not been previously reported for those mutants.

In the Calcofluor white spot-assay, 21 mutants appeared to be less sensitive than the wild type strain suggesting a possible role in chitin synthesis (Roncero *et al.*, 1988). We examined the sensitivity of these mutants to Calcofluor white in concentrations up to 500 µg/ml. Five of the mutants, for which resistance to Calcofluor white had not been reported before, could tolerate this concentration and these were therefore assayed for ChsI, ChsII and ChsIII activities (Figure 3). Chitin synthase III activity is responsible for more than 90% of the chitin in the cell wall. ChsIII is downregulated in *yjl046w*Δ, which lacks a putative protein with similarity to *Caenorhabditis elegans* lipoate-protein ligase, and in *yor*275cΔ, which lacks a putative protein with SH3 domain-binding motifs and has similarity with the pH signal transduction pathway gene *palA* of *Aspergillus nidulans*. In both mutants *in vitro* ChsI activity is also affected. Upregulation of chitin synthase III activity was found in the mutant *ynl294*Δ, which lacks an ORF containing six putative transmembrane domains and also showed a decrease in ChsI activity. Surprisingly, only this mutant showed increased sensitivity to Zymolyase, a phenotype previously observed in mutants lacking ChsIII activity (Bulawa, 1993). Thus, a clear relation between Zymolyase sensitivity, Calcofluor white resistance and chitin synthase activities could not be detected in this group of mutants. Mutants *ynl058c*Δ and *ynl233w*Δ (*bni4*Δ) had normal levels of ChsI and ChsIII activities, but in both mutants increased levels of ChsII activity were found. However, the antifungal effect of Calcofluor not only depends on its binding to cell wall chitin but also on the presence of a functional HOG pathway (Garcia-Rodriguez *et al.*, 2000). In other words, Calcofluor resistance may also be caused by the inability to respond to Calcofluor treatment.

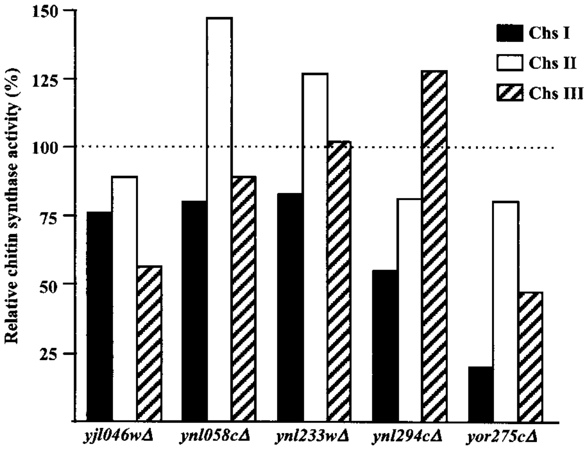

**Figure 3.** Chitin synthase activities of five mutants that are able to grow at high Calcofluor white concentration: *yjl046w*Δ, deleted in a gene homologous to a *Caenorhabditis elegans* lipoate-protein ligase gene; *ynl058c*Δ; *ynl233w*Δ (*bni4*Δ); *ynl294c*Δ; *yor275c*Δ, lacking a putative signal transduction protein. Activities are expressed as percentages of wild-type activity. Bars represent the mean values of at least two independent experiments

Copyright © 2001 John Wiley & Sons, Ltd. 

### Secondary screens to identify potential assembly enzymes

The various components of the cell wall are synthesized individually and are to a large extent processed and covalently coupled to each other at the cell surface (Figure 4A). To identify enzymes involved in processing and assembly reactions, we focused on potential cell wall-related proteins that are known to be located at the cell surface (Yeast Proteome Database: **http://www.proteome.com**, Costanzo *et al.*, 2000), proteins that show significant identity with known cell surface proteins (BLAST, **http://www.ncbi.nlm.nih.gov/BLAST**) and proteins with sequence features that are predictive of cell surface proteins (N-terminal signal sequence and/or transmembrane domains or a C-terminal GPI-anchor in combination with absence of retention signals) according to PSORT II (**http://psort.nibb.ac.jp**) analysis (Figure 1). 56 of the initially selected mutants were defective in proteins that met these criteria. To further select for mutants affected in the final cell wall construction steps, we anticipated that such mutants would secrete increased levels of cell wall components in the growth medium. Analysis of culture supernatants was performed by dot-blot immunoanalysis using antibodies directed against $\beta$1,3-glucan, $\beta$1,6-glucan (Kapteyn *et al.*, 1995), the GPI-CWPs Cwp1 (Shimoi *et al.*, 1995) and Ssr1 (Moukadiri *et al.*, 1997), and the Pir-CWP Pir2 (Russo *et al.*, 1992). An example of such an analysis is presented in Figure 4B, showing mutants *yjl062w*Δ (*gpi7*Δ/*las21*Δ) and *ydl231c*Δ with significantly increased levels and *ybr078w*Δ (*ecm33*Δ) with a slightly decreased level of Ssr1, respectively, compared to the wild type. We found that for the wild type strain the levels of $\beta$1,3-glucan, $\beta$1,6-glucan and Cwp1 are

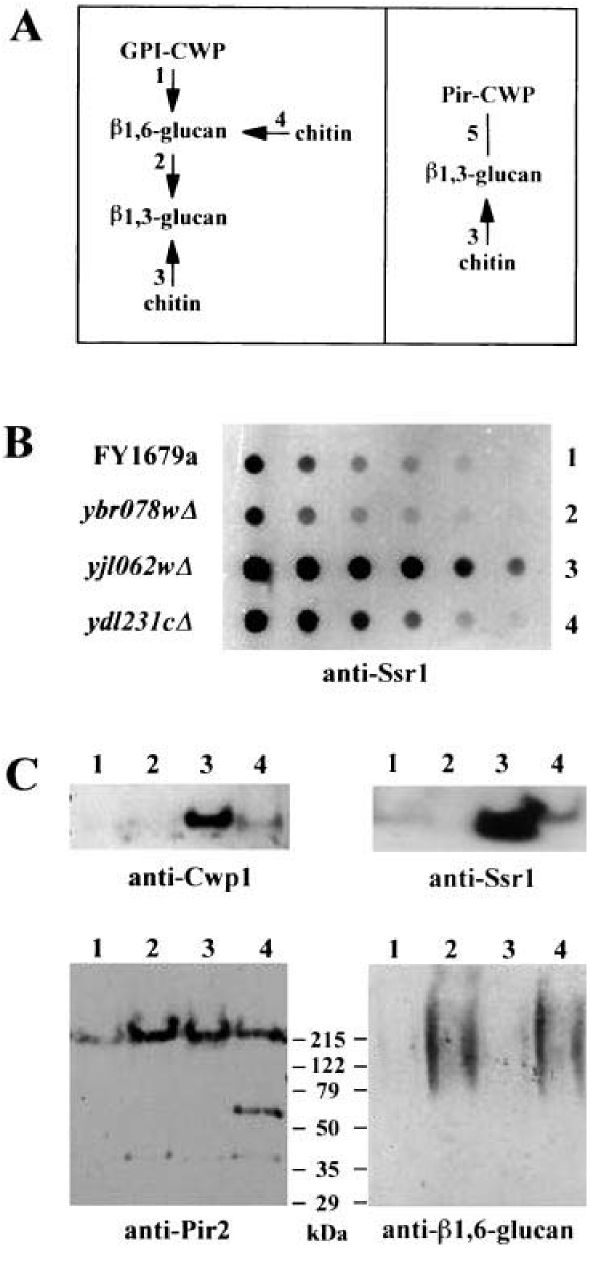

**Figure 4.** Molecular organization of the *S. cerevisiae* cell wall and immunoblot-analysis of the culture supernatant. (A) Known linkages between cell wall polymers are (1) GPI-CWPs linked to non-reducing ends of $\beta$1,6-glucan. (2) $\beta$1,6-Glucan linked to non-reducing ends of $\beta$1,3-glucan. (3) Chitin chains linked to the non-reducing ends of $\beta$1,3-glucan chains. (4) In wild type cells, a minor part of the chitin in the cell wall is bound to non-reducing ends of $\beta$1,6-glucan. (5) Pir-CWPs are linked to the $\beta$1,3-glucan chains without interconnecting $\beta$1,6-glucan but the precise linkage of Pir-CWPs to the cell wall network is unknown. (B) Dot-blot immunoanalysis of culture supernatants of putative cell wall-related mutants using anti-Ssr1 antibodies. Two-fold serial dilutions of culture supernatants were spotted; the first spots correspond to the equivalent of 400 μl culture supernatants of cells grown to $A_{600}=2$. (C) SDS-PAGE immunoblot analysis of precipitated proteins of culture supernatants of putative cell wall-related mutants with $\beta$-1,6-glucan antiserum, anti-Cwp1 antiserum, anti-Ssr1-antiserum and anti-Pir2 antiserum. Strains were grown to $A_{600}=2$ in SC medium and precipitated proteins of 200 μl culture supernatant were loaded per lane. 1, wild type strain FY1679a; 2, *ybr078w*Δ (*ecm33*Δ); 3, *yjl062w*Δ (*gpi7*Δ/*las21*Δ); 4, *ydl231c*Δ

Copyright © 2001 John Wiley & Sons, Ltd.

rather low and just above the detection limit of our immunoanalysis. We therefore focused this analysis on the identification of mutant strains that showed significant increases, rather than decreases, of cell wall components in the culture supernatant. Table 3 shows the results of this analysis for ORFs whose deletion caused an increase in the release of cell wall components to the medium suggesting a role for them in cell wall construction. Six mutants showed increased signal intensities upon analysis of culture supernatants with anti-Cwp1 and anti-Ssr1 antibodies, four of them also showing an increase upon analysis with anti-Pir2 antiserum. Some correlation between increased secretion of Cwp1 and Ssr1 in cell wall mutants was expected since these proteins both belong to the group of GPI-CWPs. On the other hand, induced expression of Cwp1, and not Ssr1, is known to be a phenotypic trait of mutants having a compromised cell wall structure (Ram *et al.*, 1998).

Pir2 is linked to $\beta$1,3-glucan independent of GPI-structures or $\beta$1,6-glucan (Figure 4A). Elevated release of Pir2 and GPI-proteins into the growth medium suggests that these mutants might have a defect in linking cell wall components to $\beta$1,3-glucan caused by a defect in either an intermolecular coupling step or remodeling of $\beta$1,3-glucan. Processing of linear $\beta$1,3-glucan is believed to create a branched molecule with acceptor sites for $\beta$1,6-glucan, chitin and Pir-proteins (Smits *et al.*, 1999). Because GPI-proteins are linked to the $\beta$1,3-glucan network via $\beta$1,6-glucan, mutants defective in the branching of $\beta$1,3-glucan are expected to also have increased levels of $\beta$1,6-glucan in the growth medium. One of the mutants displaying increased release of the three analyzed CWPs, *ydl231c*$\Delta$, deleted in an ORF specifying an unidentified putative membrane protein, indeed shows this phenotype. Alternatively, a defect in the incorporation of GPI-proteins might induce increased production of Pir proteins to compensate for lower levels of GPI-proteins in the wall. Inefficient incorporation of this Pir2 in the wall might explain the increased levels of Pir2 in the medium. This phenotype is seen in mutants *yjl062w*$\Delta$, *ybr183w*$\Delta$ (*ypc1*$\Delta$) and *ynl080c*$\Delta$. We found two genes in whose absence the culture medium became specifically enriched in Pir-cell wall proteins, suggesting

Table 3. Increased amounts of cell wall components in the culture solution of deletants that are possibly affected in cell wall assembly

| ORF | Protein name[1] | Putative location (TM)[2] | | Relative amount of cell wall component in culture solution[3] | | | | | Putative cellular role |
|---|---|---|---|---|---|---|---|---|---|
| | | | | 3G | 6G | Cwp1 | Ssr1 | Pir2 | |
| *YBR078w* | Ecm33 | PM | (0) | + | + | | | + | Cell wall remodeling[5] |
| *YBR183w* | Ypc1 | IM | (3) | | | + | + | + | Ceramide synthase activity[4] |
| *YBR207w* | Fth1 | IM | (5) | | | + | + | | Vacuolar iron transporter[4] |
| *YBR255w* | | ER | (2) | | + | | | | $\beta$1,6glucan synthesis[5] |
| *YDL231c* | | PM | (5) | | + | + | + | + | $\beta$1,3-Glucan remodeling[5] |
| *YJL062w* | Gpi7/Las21 | PM | (11) | | | + | + | + | GPI-anchor synthesis[4] |
| *YJL094c* | Kha1 | PM | (13) | + | | + | + | | Probable K+/H+-antiporter[4] |
| *YJR075w* | Hoc1 | Golgi | (2) | | | | + | + | Subunit of the Golgi mannosyltransferase complex[4] |
| *YNL080c* | | PM | (4) | | | + | + | + | GPI-protein incorporation[5] |
| *YNL159c* | | ER | (3) | + | + | | | | Cell wall remodeling[5] |
| *YNL294c* | | ER/PM | (6) | | | | | + | Pir-protein incorporation[5] |
| *YNR019w* | Are2 | IM | (8) | | + | | | + | Acyl-CoA: sterol acyltransferase activity[4] |
| *YOL092w* | | IM | (7) | | | | | + | Pir-protein incorporation[5] |

[1]YPD annotations.
[2]Cellular location and number of TransMembrane domains, experimentally determined or predicted by PSORT II. PM, Plasma membrane; ER, Endoplasmic reticulum; IM, Integral membrane; Golgi, Golgi apparatus.
[3]Determined by Western analysis using anti-$\beta$1,3-glucan (3G, only on dot-blots); anti-$\beta$1,6-glucan (6G), anti-Cwp1, anti-Ssr1 and anti-Pir2 antibodies. +, increased level of cell wall component in the culture solution of mutant relative to FY1679a.
[4]From YPD or MIPS, or [5]predicted from present data.

Copyright © 2001 John Wiley & Sons, Ltd. 

that these genes encode proteins that have a role in coupling Pir proteins to the $\beta$1,3-glucan network (Figure 4A). Yol092w is a putative transmembrane protein that belongs to a family of three members, whereas Ynl294c has no homolog in the yeast genome. In the absence of Ybr255w, which is predicted to be associated with the membrane of the endoplasmic reticulum, the levels of $\beta$1,6-glucan in the culture supernatant are increased. However the deletant does not become resistant to K1 toxin suggesting that the $\beta$1,6-glucan levels of the wall have not decreased. Possibly, the synthesis of $\beta$1,6-glucan is upregulated in this mutant. Surprisingly, the increased level of $\beta$1,6-glucan in the culture supernatant of this mutant is not accompanied by a significant increase in the GPI-cell wall proteins Cwp1 and Ssr1. This was confirmed using SDS-PAGE analysis of precipitated proteins (see below) and can not be ascribed to the presence of free $\beta$1,6-glucan in the growth medium. Likewise, mutants *ecm33*Δ and *ynl159c*Δ also have increased levels of protein-bound $\beta$1,6-glucan but no increase of Cwp1 and Ssr1, in their culture supernatants. Possibly, the bulk of $\beta$1,6-glucan in the culture supernatant of these mutants is bound to GPI-proteins other than Cwp1 and Ssr1.

Mutants showing higher levels of cell wall components in the growth medium were further analyzed by SDS-PAGE and immunodetection of proteins precipitated from the culture supernatant. For the $\beta$1,6-glucan, Cwp1, Ssr1 and Pir2 levels, this analysis generally confirmed our results obtained with the dot-blot analysis. This is exemplified in Figure 4C, showing immunoblots of three mutants, *ybr078w*Δ, *yjl062w*Δ and *ydl231c*Δ, that appear to have increased levels of different cell wall components in the growth medium compared to the wild type, as determined by dot-blot analysis (Table 3). Mutant *yjl062w*Δ releases high amounts of GPI-proteins into the culture medium whereas the amount of $\beta$1,6-glucan is similar to that of the wild type. This suggests that in this mutant coupling of GPI-proteins to $\beta$1,6-glucan is affected, which is consistent with recent studies that showed that Gpi7/Las21, like Mcd4, is involved in the addition of ethanolaminephosphate onto the core structure of GPI anchors (Flury *et al.*, 2000). Furthermore, a significant increase of Pir2 in the culture supernatant of *yjl062w*Δ was found which might be explained by increased expression of Pir-proteins as a compensation for the low amount of GPI-proteins in the cell wall. In some of the mutants, analysis with anti-Pir2 antibodies revealed that in addition to Pir2 ($\pm$220 kDa) a weak signal at about 40 kDa was detected. This protein may correspond to Pir4/Cis3 which immunoreacted with polyclonal anti-Pir2 antibodies (Kapteyn *et al.*, 1999). For *ydl231c*Δ, an additional protein band of about 60 kDa is observed, that is postulated to be a processed form of the Pir2 protein (Kapteyn *et al.*, 1999).

None of the mutants showing immunoreactivity in the dot-blots with the anti-$\beta$1,3-glucan antibodies (Table 3) gave rise to clear signals by immunoanalysis of precipitated medium proteins (not shown). This indicates that $\beta$1,3-glucan molecules detected in the medium are not linked to protein.

### Secondary screens to identify potential cell wall regulatory proteins

To identify proteins directly affecting the cell wall integrity pathway and proteins that are otherwise involved in regulation of cell wall formation, the 145 initially selected mutants were analyzed for sensitivity to calcium, sensitivity to caffeine, osmotic remediability of caffeine hypersensitivity using sorbitol, and Slt2/Mpk1 MAP kinase activation (Figure 1).

Intracellular calcium levels are thought to influence cell morphogenesis and cell wall biosynthesis in several ways. For instance, calcium is likely to be involved in the regulation of actin-dependent morphogenesis via Cdc24 (Miyamoto *et al.*, 1991), in polarized secretion via calmodulin (Peters and Mayer, 1998) and in regulation of glucan synthesis via calcineurin (Zhao *et al.*, 1998). We therefore speculated that many of the mutants might be hypersensitive to the presence of calcium in the growth medium. The results of the calcium sensitivity test show that 24% of the mutants analyzed were indeed sensitive to calcium (but not pleiotropically hypersensitive to high salinity). Conversely, most of the calcium-hypersensitive mutants (92%) had additional phenotypes related to defects in cell wall formation. There does not seem to be a strong correlation between altered morphology and calcium hypersensitivity since only 22% of the morphological mutants were hypersensitive to calcium. However, a high proportion of mutants with decreased glucan synthase activity were also more sensitive to calcium (Table 2).

Copyright © 2001 John Wiley & Sons, Ltd. 

Caffeine, besides being a well-known inhibitor of cAMP phosphodiesterase, has also been found to stimulate dual phosphorylation of Slt2, the MAP kinase component of the cell wall integrity signal transduction pathway (Martín *et al.*, 2000). Mutants involved in the cell wall integrity pathway are more sensitive to caffeine, displaying a lytic phenotype in the presence of this compound that can be prevented by osmotic stabilization (Martín *et al.*, 1996). 71 of the 145 analyzed mutants appeared caffeine-sensitive but only 17 of them were rescued by the addition of sorbitol suggesting that these osmotically-remedial mutants have cell wall defects that are due to a failure in the regulation of the cell wall integrity by this pathway. Eleven of the seventeen osmotically stabilized mutants had altered morphologies (Table 4), which is consistent with the notion that the cell wall integrity pathway is involved in coordinating cell wall synthesis with the cell cycle and morphogenetic events (Igual *et al.*, 1996; Gray *et al.*, 1997).

More direct information on activation of the Pkc1-Slt2-pathway was obtained by measuring the levels of the activated form of the MAP kinase Slt2. Mutations in genes involved in positive modulation of the pathway are expected to fail in activating the pathway whereas the absence of negative modulators should result in activation of the pathway even in the absence of stimulating signals. On the other hand, this pathway is activated in response to environmental conditions that jeopardize cell wall stability in order to induce a cell wall compensation mechanism (Jung and Levin, 1999; De Nobel *et al.*, 2000). Constitutive activation of Slt2 has been also seen in mutants affected in cell wall functions, such as *kre9*Δ, *gas1*Δ and *fks1*Δ, in response to the cell wall defects displayed by these mutants (De Nobel *et al.*, 2000). The 145 initially selected mutants were analyzed by Western analysis using antibodies raised against the dually phosphorylated region (Thr202/Tyr204) of p44/42 MAP kinases, which have been shown to recognize the active form of the yeast Slt2 MAP kinase (Martín *et al.*, 2000). Compared to wild-type, fourteen mutants showed elevated levels of Slt2 phosphorylation at 24°C, indicative of proteins that directly regulate the activity of the pathway or proteins whose absence triggers a constitutive activation of the cell wall integrity pathway as a consequence of a weakened cell wall (Table 5). Interestingly, among these

Table 4. Caffeine-hypersensitive mutants osmotically stabilized by sorbitol

| ORF | Protein name[1] | Homology with[1] | Altered morphology[2] | Putative biochemical function or cellular role[3] |
|---|---|---|---|---|
| *YBR078w* | Ecm33 | | R, L, EFL | Cell wall maintenance |
| *YBR133c* | Hsl7 | | HP | Cell cycle control |
| *YDL149w* | Apg9 | | L | Vesicular transport |
| *YGL197w* | Mds3 | | I, EFL | Transcription factor |
| *YGR200c* | Elp2 | | | Polymerase II transcription |
| *YGR275w* | Rtt102 | | I, HP | Regulator of Ty1 transposition |
| *YJL056c* | Zap1 | | | Transcription factor |
| *YJL204c* | | Tor2 | HP | Vesicular transport |
| *YJR013w* | | Human angiotensin II type 1b receptor | L | Unknown |
| *YNL166c* | Bni5 | | L, CS, EFL | Unknown |
| *YNL214w* | Pex17 | | L | Peroxisome receptor |
| *YNL288w* | | | | Unknown |
| *YNL325c* | Fig4 | | L | Small molecule transport |
| *YNR019w* | Are2 | | I | Acyl-CoA : sterol acyltransferase |
| *YOL018c* | Tlg2 | | | Vesicular transport |
| *YOL036w* | | | | Unknown |
| *YOL092w* | | Ybr147p | | Unknown |

[1]Protein names following YPD annotations.
[2]Microscopic observations of Calcofluor white-stained cells. CS, chitin spots; EFL, enhanced fluorescence; HP, hyperpolarized growth; I, irregular morphology; L, large cells; R, rounded cells.
[3]From YPD or MIPS.

Copyright © 2001 John Wiley & Sons, Ltd. 

Table 5. Deletants with increased levels of dually phosphorylated MAP kinase Slt2

| ORF | Protein name[1] | Homology with[1] | Relative Slt2 phosphorylation[2] 24°C | 39°C | Other relevant phenotypes[3] Caf-S | Sorb-rem | Putative biochemical function (cellular role)[4] |
|---|---|---|---|---|---|---|---|
| *YGL131c* | | Set1 | + | | no | no | Putative transcription factor |
| *YJL056c* | Zap1 | | + | | yes | yes | Transcription factor (metalloregulation) |
| *YNL227c* | | DNA-J like proteins | + | + | yes | no | Chaperone (protein folding; putative transcription factor) |
| *YGL128c* | | DNA-J like proteins | + | + | yes | no | Chaperone (protein folding) |
| *YGL099w* | | Human Hsr1 | + | + | yes | no | GTP-binding protein (chromatin structure) |
| *YOL115w* | Trf4 | | + | | yes | no | DNA repair (chromatin structure) |
| *YJR059w* | Ptk2 | Hal5 | + | | no | no | Protein kinase (small molecule transport) |
| *YNL101w* | | MFS proteins | + | | yes | no | Transporter (small molecule transport) |
| *YBR078w* | Ecm33 | | + | | yes | yes | Unknown (cell wall maintenance) |
| *YBR229c* | Rot2 | | + | + | no | no | Glucosidase II (cell wall maintenance) |
| *YNL059c* | Arp5 | | + | | yes | no | Actin-related protein |
| *YBL006c* | | | + | | yes | no | Unknown |
| *YBR266c* | | | + | + | no | no | Unknown |
| *YGL133w* | | | + | | yes | no | Unknown |

[1]Protein names following YPD annotations.
[2]Determined by Western analysis using anti-phospho-p44/42 MAPK antibodies. +, increased phosphorylation in mutant relative to FY1679a analyzed under the same conditions.
[3]Caf-S, Caffeine hypersensitive; Sorb-rem, sorbitol-remediable.
[4]From YPD or MIPS, or homology search by BLAST.

mutants are mutants that are deleted in ORFs specifying putative transcription factors, typical signaling molecules (a GTP-binding protein and a protein kinase), chaperones and proteins involved in cell wall maintenance. In wild-type cells that are incubated at 39°C, an increased amount of the cellular Slt2 becomes phosphorylated (Martín *et al.*, 2000). After 2 h of growth at 39°C, five of the 14 cell wall-related mutants with elevated Slt2 phosporylation at 24°C showed increased Slt2 activation compared to the wild type grown at high temperature (Table 5). Two of the mutants displaying this phenotype are lacking in putative members of the DNAJ molecular chaperones. This class of proteins, usually induced by heat shock and other environmental stresses, play a role in protecting cells from these adverse conditions. Increased activation of Slt2 at 24°C but not at 39°C, as observed for the other nine mutants, suggests that these proteins might be involved in maintaining Slt2 activity at 24°C at a low basal level. An example of the MAP kinase phosphorylation assay is presented in Figure 5, including the *yjl056c*Δ (*zap1*Δ) strain, which shows increased activation at 24°C, and the *ygl128c*Δ strain, in which hyperactivation of the pathway occurs both under inducing (39°C) and non-inducing conditions (24°C).

Mutants showing decreased Slt2 phosphorylation at 39°C were not detected, indicating that none of the mutations studied is indispenable for heat-shock induced activation of the Pkc1-Slt2 pathway.

### Discussion

### General approach

We have developed a hierarchical approach to identify and classify genes involved in cell wall

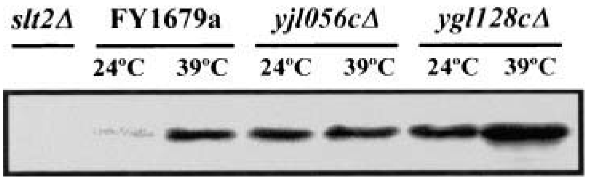

**Figure 5.** Anti-phospho-Slt2 immunoblot analysis of 80 μg protein extracts obtained from the mutant strains *yjl056c*Δ and *ygl128c*Δ, and the isogenic wild type strain FY1679a and the mutant strain FYDK (*slt2*Δ) as a control. Cells were cultured to mid-log phase at 24°C and, where indicated, shifted to 39°C for 2 h prior to collection. Anti-Slt2 immunoblot analysis was performed on the same membrane to verify that similar amounts of Slt2 were present in every lane (data not shown)

Copyright © 2001 John Wiley & Sons, Ltd.

dynamics. In the present work, the complete EUROFAN set of 620 mutants bearing deletions in non-essential genes was screened using a few simple tests that are indicative of cell wall defects. We used the following primary screens, namely altered sensitivity to Calcofluor white, SDS, and sonication, and altered morphology. Initially, we also investigated whether additional cell wall mutants could be identified by screening for cell lysis at 37°C. However, analysis of a subset of the EUROFAN mutants for this phenotype (data not shown) indicated that, although being selective for mutants with altered cell integrity, this test did not result in the identification of additional mutants and studying cell lysis at 37°C as a primary test was therefore not continued. Mutants selected with our primary screens (145 or 23%) were analyzed further with tests of increasing specificity to detect genes involved in specific aspects of cell wall formation or in regulatory signaling pathways. These secondary screens revealed that 91% of the mutants identified by the Calcofluor white assay, 98% of the mutants identified by the SDS test and 86% of the mutants identified by sonication have additional cell wall-related phenotypes. In accordance with the close relationship existing between the formation of a functional cell wall and morphogenesis, additional phenotypes were found for 94% of the morphological mutants. Thus, all the primary screens used in this work are indeed strongly selective for mutants with an altered cell wall. Except for the mentioned correlation between the mutants selected by the sonication assay and those identified by microscopic observation, our primary screens showed limited overlap indicating that they were largely independent from each other.

Altogether, for 131 (90%) of the mutants identified in the first round, additional cell wall-related phenotypes were found, indicating that the number of potentially false positives was acceptable and that our screens were efficient. Extrapolated for the entire yeast genome containing more than 6000 genes, our data suggest that at least 1200 genes directly or indirectly affect the cell wall. However, because this study focussed on non-essential gene deletants only, the actual number of genes affecting cell wall biosynthesis is likely to be even higher. Using constructs in which genes of interest are cloned behind a doxycycline-repressible promoter, we are currently applying the same approach to study the involvement of essential genes in cell wall biosynthesis (not shown). Furthermore, as is inherent to this type of approach, our screens did not identify all cell wall-related genes. It is known that, due to redundancy, deletion of some individual genes encoding cell wall proteins do not cause clear phenotypes (Mrsa *et al.*, 1997; Rodriguez-Peña *et al.*, 2000; Lussier *et al.*, 1997). In fact, some known cell wall-related genes in the EUROFAN collection (*GAS4*, *CRH1* and *PST1*) not selected by our screens, belong to gene families. Finally, our screening results show that genes performing a wide range of cellular functions, ranging from transcription, translation, metabolism, or having a mitochondrial function are linked to cell wall metabolism (Table 6). This points to the existence of regulatory associations between apparently unrelated cellular processes. Genome-wide screens such as the one presented here will be very powerful tools to uncover such associations.

### Cell wall synthesis and cell wall assembly enzymes

26 mutants showed altered $\beta$1,3-glucan or chitin synthetic activities indicating that the corresponding proteins may be involved in the synthesis of these cell wall polymers. They could be directly modulating the activity of the synthase complexes or affecting transport of components of the synthetic machinery. For example, two of the mutants displaying a diminished glucan synthase activity (Table 2) are defective in proteins involved in the regulation of protein trafficking to the membrane: Vps53 (Yjl029c) whose role in protein sorting at the late Golgi compartment has been recently described (Conibear and Stevens, 2000) and the syntaxin Tlg2 (Yol018c). A related member of the syntaxin family, Tlg1, has been reported to be required for the correct localization of chitin synthase III by mediating trafficking of this enzyme to polarized growth sites (Holthuis *et al.*, 1998). The cyclosporin hypersensitivity displayed by the *vps53*Δ mutant is consistent with the presumed role of the corresponding protein in glucan biogenesis. Furthermore, a strong correlation with calcium hypersensitivity was observed among the mutants with decreased $\beta$1,3-glucan synthase activity, reinforcing the idea of the involvement of calcium in the regulation of the synthesis of this polymer.

In addition, a role in the regulation of the expression of biosynthetic enzymes can not be

Copyright © 2001 John Wiley & Sons, Ltd.

Table 6. Genes identified in the primary screening. Classification according to MIPS and YPD databases

| | | |
|---|---|---|
| Unknown genes | *YBL006c, YBL009w, YBL029w, YBR016w, YBR071w, YBR175w, YBR255w, YBR258c, YBR266c, YDL074c (BRE1), YDL115c, YDL117w, YDL124w, YDL146w, YDL158c, YDL231c, YDR015c, YDR065w, YDR067c, YDR071c, YGL114w, YGL131c, YGL133w (ITC1), YGL138c, YGR210c, YGR275w (RTT102), YJL004c (SYS1), YJL018w, YJL019w, YJL070c, YJL149w, YJR001w, YJR013w, YJR044c, YJR070c, YLL044w, YLL057c, YLR023c, YNL058c, YNL080c, YNL119w, YNL134c, YNL159c, YNL177c, YNL213c, YNL215w (IES2), YNL224c, YNL278w (CAF120), YNL288w (CAF40), YNL294c, YNL323w, YNR047w, YNR048w, YNR051c (BRE5), YNR068c, YOL036w, YOL072w (THP1), YOL092w, YOL093w, YOL124c, YOL138c, YOR007c (SGT2), YOR275c, YOR279c, YOR322c* | 65 (44.8%) |
| Genes previously related to cell wall biogenesis and maintenance | *YBR078w (ECM33), YBR229c (ROT2), YGR216c (GPI1), YJL062w (LAS21/GPI7), YJR075w (HOC1), YNL095c (h ECM3), YNL233w (BNI4), YNL106c (INP52), YNR030w (ECM39)* | 9 (6.2%) |
| Genes previously related to morphogenesis | *YBR133c (HSL7), YDL225w (SEP7), YGR262c (BUD32), YJR053w (BFA1), YLR074c (BUD20), YNL059c (ARP5), YNL166c (BNI5), YNL223w (AUT2), YNL225c (CNM67)* | 9 (6.2%) |
| Genes not previously related to the cell wall | *YBL024w (NCL1), YBL025w (RRN10), YBL067c (UBP13), YBR044c (TCM62), YBR183w (YPC1), YBR207w (FTH1), YBR264c (YPT10), YDL005c (MED2), YDL059c (RAD59), YDL077c (VAM6), YDL112w (TRM3), YDL149w (APG9), YDL167c (NRP1), YDL171c (GLT1), YDL243c (AAD4), YDR073w (SNF11), YGL012w (ERG4), YGL078c (DBP3), YGL100w (SEH1), YGL197w (MDS3), YGR200c (ELP2), YJL006c (CTK2), YJL029c (VPS53), YJL047c (RTT101), YJL056c (ZAP1), YJL071w (ARG2), YJL094c (KHA1), YJL124c (LSM1), YJL134w (LCB3), YJL204c (RCY1), YJR043c (POL32), YJR059w (PTK2), YJR074w (MOG1), YLL040c (VPS13), YLR024c (UBR2), YLR070c (XYL2), YNL054w (VAC7), YNL097c (PHO23), YNL214w (PEX17), YNL304w (YPT11), YNL306w (YMS18), YNL309w (STB1), YNL321w, YNL325c (FIG4), YNR019w (ARE2), YOL018c (TLG2), YOL115w (TRF4), YOL151w (GRE2), YOR162c (YRR1)* | 49 (33.8%) |
| Genes homologous to genes not previously related to the cell wall | *YBL051c, YBR162c (TOS1), YDL100c, YDL125c (HNT1), YGL099w, YGL128c, YJL046w, YJL065c, YLR114c (EFR4), YNL101w, YNL107w (YAF9), YNL227c, YOL152w (FRE7)* | 13 (9.0%) |
| | | 145 (100%) |

ruled out for proteins whose absence leads to altered synthesis of cell wall polysaccharides. Transcription of *CHS3* and *FKS1*, the genes encoding chitin synthase III, and a catalytic subunit of the $\beta$1,3-glucan synthase complex, respectively, was found to be regulated by the cell integrity pathway (Igual *et al.*, 1996; Jung and Levin, 1999). Moreover, an increase in the chitin content of the cell wall has been proposed to be part of a set of reactions induced in response to cell wall perturbation (Popolo *et al.*, 1997; Ram *et al.*, 1998). Therefore, altered biosynthesis of cell wall polymers in some of these mutants may be a consequence of compensation mechanisms triggered by a defective cell wall. For example, deletion of *YNL058c*, an ORF whose transcription is also regulated by this pathway (Jung and Levin, 1999), led to increased chitin synthase II activity. Additionally, mutant *yor275*$\Delta$, displaying a significant reduction both in chitin synthase I and III, is defective in a protein that has similarity to Bro1, which has been reported to interact with components of the cell integrity pathway (Nickas and Yaffe, 1996).

Our knowledge of enzymes involved in cell wall assembly is still limited. To identify candidate enzymes involved in cell wall construction steps, we inferred that mutants lacking such enzymes release increased amounts of cell wall components to the growth media. Examples of genes whose deletion causes such phenotypes are *GAS1*, which is involved in processing of short linear $\beta$1,3-glucan chains (Mouyna *et al.*, 2000), and *MCD4* which is involved in transfer of ethanolaminephosphate onto the core structure of GPI anchors (Gaynor *et al.*, 1999). Mutants lacking these genes secrete high amounts of cell wall proteins in the culture media (Ram *et al.*, 1998; Gaynor *et al.*, 1999). In agreement with this, we found high amounts of the GPI-proteins Ssr1 and Cwp1 in the culture supernantant of the mutant deleted in *YJL062w* (*GPI7/LAS21*) which belongs to the *MCD4* gene family (Flury *et al.*, 2000). Immunoanalysis of culture

Copyright © 2001 John Wiley & Sons, Ltd. 

supernatants revealed, among others, candidate genes involved in coupling of Pir-proteins to the $\beta$1,3-glucan network (*YNL294c* and *YOL092w*), in $\beta$1,3-glucan remodeling (*YBR078w*, *YDL231c* and *YNL159c*) and in GPI anchor biosynthesis (*YJL062w, YNL080c*). This method therefore proved to be an effective tool to identify proteins involved in assembly of cell wall components.

### Regulation of cell wall synthesis

The activation of the cell integrity pathway has been also studied in the selected 145 mutants. Two classes of mutants with altered activation of this pathway were expected. First, mutants lacking components or modulators of the regulatory signaling pathway and, second, mutants which are able to trigger the compensatory mechanism mediated by this pathway because they are defective in proteins playing an important role in cell wall formation. In agreement with this, typical signaling proteins and proteins known to be involved in cell wall biogenesis and maintenance together with other proteins of unknown function were identified by this screen. Most of the mutants displaying a constitutive activation of the cell integrity pathway were also more sensitive to caffeine, although this phenotype was osmotically remediable for only two of them. In total, 17 mutants showed caffeine hypersensitivity which could be stabilized by sorbitol. Among them, 15 mutants did not show an altered pattern of MAP kinase Slt2 activation, suggesting that they have alterations in the cell wall that do not constitutively activate the cell integrity pathway.

### Concluding remarks

The use of the hierarchical approach described here has the advantage that a large collection of mutants can be screened rapidly using a few tests that are indicative of putative cell wall defects. A total of 65 genes with no previously experimentally-proved or predicted function have been identified among the EUROFAN collection as having cell wall-related phenotypes (Table 6). Based on previous phenotypic analysis of mutants or by database analysis of protein sequences, a number of proteins among the EUROFAN collection had been suggested to be related to cell wall biogenesis or maintenance. Nine of these, among which Ecm33, Rot2 and Gpi7, were also identified by our screening procedure. Additionally, nine genes were selected that had previously been related to morphogenesis (Table 6). In conclusion, the systematic phenotypic screening of deletion mutants has proved to be a powerful tool in selecting a wide range of mutants with cell wall-related phenotypes of *S. cerevisiae* and should also be applicable to related fungi having similar cell wall organizations, such as *Candida albicans* (Kapteyn *et al.*, 2000)

### Acknowledgements

The work described in this article was part of the EUROFAN II program funded by the European Union and co-financed by grants BIO99-1384-CE and BIOg8-1516-CE from Comisión Interministerial de Ciencia y Tecnología (CICYT, Spain). We thank Stephan Brekelmans, Margarita Arroyo, Juan C. Ribas, Cesar Roncero, Luis J. García, Miguel Sánchez and Mercedes Pardo for technical assistance and critical discussion of this work, and Amalia Vázquez and Alberto Alvarez from Centro de Citometría de Flujo (Universidad Complutense, Spain) for flow cytometry analysis. R. Wickner, T. Watanabe and M. Schmitt (NIH, Bethesda, USA) are thanked for providing killer toxin producing strains. J.M.R. and C.R. were recipients of a postdoctoral and a predoctoral fellowship, respectively, from the Consejería de Educación y Cultura de la Comunidad de Madrid (Spain). We thank Eli Lilly (Indianapolis, USA) for kindly providing Echinocandin B.

### References

Bickle M, Delley PA, Schmidt A, Hall MN. 1998. Cell wall integrity modulates *RHO1* activity via the exchange factor *ROM2*. *EMBO J* **17**: 2235–4225.

BLAST. **http://www.ncbi.nlm.nih.gov/BLAST**

Boone C, Sommer SS, Hensel A, Bussey H. 1990. Yeast *KRE* genes provide evidence for a pathway of cell wall $\beta$-glucan assembly. *J Cell Biol* **110**: 1833–1843.

Bulawa CE. 1993. Genetics and molecular biology of chitin synthesis in fungi. *Review Annu Rev Microbiol* **47**: 505–534.

Choi WJ, Cabib E. 1994. The use of divalent cations and pH for the determination of specific yeast chitin synthetases. *Anal Biochem* **219**: 368–372.

Cid VJ, Duran A, Del Rey F, Snyder MP, Nombela C, Sánchez M. 1995. Molecular basis of cell integrity and morphogenesis in *Saccharomyces cerevisiae*. *Microbiol Rev* **59**: 345–386.

Cid VJ, Cenamor R, Sánchez M, Nombela C. 1998. A mutation in the Rho1-GAP-encoding gene *BEM2* from *Saccharomyces cerevisiae* affects morphogenesis and cell wall functionality. *Microbiology* **144**: 25–36.

Conibear E, Stevens TH. 2000. Vps52p, Vps53p, and Vps54p form a novel multisubunit complex required for protein sorting at the yeast late Golgi. *Mol Biol Cell* **11**: 305–323.

Costanzo MC, Hogan JD, Cusick ME, *et al.* 2000. The Yeast Proteome Database (YPD) and *Caenorhabditis elegans* Proteome Database (WormPD): comprehensive resources for

Copyright © 2001 John Wiley & Sons, Ltd. 

the organization and comparison of model organism protein information. *Nucleic Acids Res* **28**: 73–76. YPD: **http://www.proteome.com**

Dallies N, Francois J, Paquet V. 1998. A new method for quantitative determination of polysaccharides in the yeast cell wall. *Yeast* **14**: 1297–1306.

De Nobel JG, Klis FM, Ram A, Van Unen H, Priem J, Munnik T, Van Den Ende H. 1991. Cyclic variations in the permeability of the cell wall of *Saccharomyces cerevisiae*. *Yeast* **7**: 589–598.

De Nobel H, Ruiz C, Martín H, *et al.* 2000. Cell wall perturbation in yeast results in dual phosphorylation of the Slt2/Mpk1 MAP kinase and in an Slt2-mediated increase in *FKS2-lacZ* expression, glucanase resistance and thermotolerance. *Microbiol* **146**: 2121–2132.

Elorza MV, Rico H, Sentandreu R. 1983. Calcofluor white alters the assembly of chitin fibrils in *Saccharomyces cerevisiae* and *Candida albicans* cells. *J Gen Microbiol* **129**: 1577–1582.

Flury I, Benachour A, Conzelmann A. 2000. YLL031c belongs to a novel family of membrane proteins involved in the transfer of ethanolaminephosphate onto the core structure of glycosylphosphatidylinositol anchors in yeast. *J Biol Chem* **275**: 24458–24465.

Font de Mora J, Herrero E, Sentandreu R. 1993. A kinetic study on the regeneration of *Candida albicans* protoplasts in the presence of cell wall inhibitors. *FEMS Microbiol Lett* **111**: 43–47.

Garcia-Rodriguez LJ, Duran A, Roncero C. 2000. Calcofluor antifungal action depends on chitin and a functional high-osmolarity glycerol response (HOG) pathway: evidence for a physiological role of the *Saccharomyces cerevisiae* HOG pathway under noninducing conditions. *J Bacteriol* **182**: 2428–2437.

Gaynor EC, Mondesert G, Grimme SJ, Reed SI, Orlean P, Emr SD. 1999. *MCD4* encodes a conserved endoplasmic reticulum membrane protein essential for glycosylphosphatidylinositol anchor synthesis in yeast. *Mol Biol Cell* **10**: 627–648.

Gray JV, Ogas JP, Kamada Y, Stone M, Levin DE, Herskowitz I. 1997. A role for the Pkc1 MAP kinase pathway of *Saccharomyces cerevisiae* in bud emergence and identification of a putative upstream regulator. *EMBO J* **16**: 4924–4937.

Holthuis JCM, Nichols BJ, Pelham HRB. 1998. The syntaxin Tlg1p mediates trafficking of chitin synthase III to polarized growth sites in yeast. *Mol Biol Cell* **9**: 3383–3397.

Igual JC, Johnson AL, Johnston LH. 1996. Coordinated regulation of gene expression by the cell cycle transcription factor Swi4 and the protein kinase C MAP kinase pathway for yeast cell integrity. *EMBO J* **15**: 5001–5013.

Ishiguro J, Saitou A, Duran A, Ribas JC. 1997. *cps1+*, a *Schizosaccharomyces pombe* gene homolog of *Saccharomyces cerevisiae FKS* genes whose mutation hypersensitivity to cyclosporin A and papulacandin B. *J Bacteriol* **179**: 7653–7662.

Jung US, Levin DE. 1999. Genome-wide analysis of gene expression regulated by the yeast cell wall integrity signalling pathway. *Mol Microbiol* **34**: 1049–1057.

Kapteyn JC, Montijn RC, Dijkgraaf GJP, Van Den Ende H, Klis FM. 1995. Covalent association of $\beta$-1,3-glucan with $\beta$-1,6-glucosylated mannoproteins in cell walls of *Candida albicans*. *J Bacteriol* **177**: 3788–3792.

Kapteyn JC, Ram AF, Groos EM, *et al.* 1997. Altered extent of cross-linking of $\beta$-1,6-glucosylated mannoproteins to chitin in *Saccharomyces cerevisiae* mutants with reduced cell wall $\beta$-1,3-glucan content. *J Bacteriol* **179**: 6279–6284.

Kapteyn JC, Van Egmond P, Sievi E, Van Den Ende H, Makarow M, Klis FM. 1999. The contribution of the O-glycosylated protein Pir2p/Hsp150 to the construction of the yeast cell wall in wild-type cells and $\beta$1,6-glucan-deficient mutants. *Mol Microbiol* **31**: 1835–1844.

Kapteyn JC, Hoyer LL, Hecht JE, *et al.* 2000. The cell wall architecture of *Candida albicans* wild-type cells and cell wall-defective mutants. *Mol Microbiol* **35**: 601–611.

Kasahara S, Ben Inoue S, Mio T, *et al.* 1994. Involvement of cell wall beta-glucan in the action of HM-1 killer toxin. *FEBS Lett* **348**: 27–32.

Ketela T, Green R, Bussey H. 1999. *Saccharomyces cerevisiae* Mid2p is a potential cell wall stress sensor and upstream activator of the PKC1-MPK1 cell integrity pathway. *J Bacteriol* **181**: 3330–3340.

Klis FM. 1994. Review: cell wall assembly in yeast. *Yeast* **10**: 851–869.

Klis FM, Ram AFJ, Montijn RC, *et al.* 1998. Posttranslational modifications of secretory proteins. *Methods Microbiol* **26**: 223–238.

Kollár R, Petrakova E, Ashwell G, Robbins PW, Cabib E. 1995. Architecture of the yeast cell wall. The linkage between chitin and beta(1-->3)-glucan. *J Biol Chem* **270**: 1170–1178.

Kollár R, Reinhold BB, Petrakova E, *et al.* 1997. Architecture of the yeast cell wall. Beta(1-->6)-glucan interconnects mannoprotein, beta(1-->)3-glucan, and chitin. *J Biol Chem* **272**: 17762–17775.

Lussier M, White A-M, Sheraton J, *et al.* 1997. Large scale identification of genes involved in cell surface biosynthesis and architecture in *Saccharomyces cerevisiae*. *Genetics* **147**: 435–450.

Martín H, Arroyo J, Sanchez M, Molina M, Nombela C. 1993. Activity of the yeast MAP kinase homologue Slt2 is critically required for cell integrity at 37°C. *Mol Gen Genet* **241**: 177–184.

Martín H, Castellanos MC, Cenamor R, Sanchez M, Molina M, Nombela C. 1996. Molecular and functional characterization of a mutant allele of the mitogen-activated protein-kinase gene *SLT2* (*MPK1*) rescued from yeast autolytic mutants. *Curr Genet* **29**: 516–522.

Martín H, Rodriguez-Pachon JM, Ruiz C, Nombela C, Molina M. 2000. Regulatory mechanisms for modulation of signaling through the cell integrity Slt2-mediated pathway in *Saccharomyces cerevisiae*. *J Biol Chem* **275**: 1511–1519.

Mazur P, Morin N, Baginsky W, *et al.* 1995. Differential expression and function of two homologous subunits of yeast 1,3-beta-D-glucan synthase. *Mol Cell Biol* **15**: 5671–5681.

MIPS. **http://www.mips.biochem.mpg.de/proj/eurofan/eurofan_2/**

Miyamoto S, Ohya Y, Sano Y, Sakaguchi S, Iida H, Anraku Y. 1991. A *DBL*-homologous region of the yeast *CLS4/CDC24* gene product is important for $Ca^{2+}$-modulated bud assembly. *Biochem Biophys Res Commun* **181**: 604–610.

Montijn RC, Van Rinsum J, Van Schagen FA, Klis FM. 1994. Glucomannoproteins in the cell wall of *Saccharomyces cerevisiae* contain a novel type of carbohydrate side chain. *J Biol Chem* **269**: 19338–19342.

Moukadiri I, Armero J, Abad A, Sentandreu R, Zueco J. 1997. Identification of a mannoprotein present in the inner layer of

Copyright © 2001 John Wiley & Sons, Ltd. 

the cell wall of *Saccharomyces cerevisiae*. *J Bacteriol* **179**: 2154–2162.

Mouyna I, Fontaine T, Vai M, *et al.* 2000. Glycosyl phosphatidylinositol-anchored glucanosyltransferases play an active role in the biosynthesisof the fungal cell wall. *J Biol Chem* **275**: 14882–14889.

Mrsa V, Seidl T, Gentzsch M, Tanner W. 1997. Specific labelling of cell wall proteins by biotinylation. Identification of four covalently linked *O*-mannosylated proteins of *Saccharomyces cerevisiae*. *Yeast* **13**: 1145–1154.

Nickas ME, Yaffe MP. 1996. *BRO1*, a novel gene that interacts with components of the Pkc1p-mitogen-activated protein kinase pathway in *Saccharomyces cerevisiae*. *Mol Cell Biol* **16**: 2585–2593.

Oliver S. 1996. A network approach to the systematic analysis of yeast gene function. *Trends Genet* **12**: 241–242.

Ovalle R, Lim ST, Holder B, Jue CK, Moore CW, Lipke PN. 1998. A spheroplast rateassay for determination of cell wall integrity in yeast. *Yeast* **14**: 1159–1166.

Peters C, Mayer A. 1998. Ca2+/calmodulin signals the completion of docking and triggers a late step of vacuole fusion. *Nature* **396**: 575–580.

Popolo L, Vai M. 1999. The Gas1 glycoprotein, a putative wall polymer cross-linker. *Biochim Biophys Acta* **1426**: 385–400.

Popolo L, Gilardelli D, Bonfante P, Vai M. 1997. Increase in chitin as an essential response to defects in assembly of cell wall polymers in the *ggp1Δ* mutant of *Saccharomyces cerevisiae*. *J Bacteriol* **179**: 463–469.

Pringle JR, Preston RA, Adams AE, *et al.* 1989. Fluorescence microscopy methods for yeast. *Methods Cell Biol* **31**: 357–435.

Pringle JR. 1991. Staining of bud scars and other cell wall chitin with calcofluor. *Methods Enzymol* **194**: 732–735.

PSORT II. **http://psort.nibb.ac.jp**

Ram AFJ, Wolters A, Ten Hoopen R, Klis FM. 1994. A new approach for isolating cell wall mutants in *Saccharomyces cerevisiae* by screening for hypersensitivity to Calcofluor White. *Yeast* **10**: 1019–1030.

Ram AF, Kapteyn JC, Montijn RC, *et al.* 1998. Loss of the plasma membrane-bound protein Gas1p in *Saccharomyces cerevisiae* results in the release of $\beta$-1,3-glucan into the medium and induces a compensation mechanism to ensure cell wall integrity. *J Bacteriol* **180**: 1418–1424.

Rodriguez-Peña JM, Cid VJ, Arroyo J, Nombela C. 2000. A novel family of cell wall-related proteins regulated differently during the yeast life cycle. *Mol Cell Biol* **20**: 3245–3255.

Roncero C, Duran AJ. 1985. Effect of Calcofluor white and Congo red on fungal cell wall morphogenesis: *in vivo* activation of chitin polymerization. *J Bacteriol* **163**: 1180–1185.

Roncero C, Valdivieso MH, Ribas JC, Duran A. 1988. Effect of calcofluor white on chitin synthases from *Saccharomyces cerevisiae*. *J Bacteriol* **170**: 1945–1949.

Ruiz C, Cid VJ, Lussier M, Molina M, Nombela C. 1999. A large-scale sonication assay for cell wall mutant analysis in yeast. *Yeast* **15**: 1001–1008.

Russo P, Kalkkinen N, Sareneva H, Paakkole J, Makarow M. 1992. A heat shock protein from *Saccharomyces cerevisiae* encoding a secretory glycoprotein. *Proc Natl Acad Sci U S A* **89**: 3671–3675.

Schmitt M, Radler F. 1987. Mannoprotein of the yeast cell wall as primary receptor for the killer toxin of *Saccharomyces cerevisiae* strain 28. *J Gen Microbiol* **133**: 3347–3354.

Shahinian S, Bussey H. 2000. $\beta$-1,6-Glucan synthesis in *Saccharomyces cerevisiae*. *Mol Microbiol* **35**: 477–489.

Shimoi H, Iimura Y, Obata T. 1995. Molecular cloning of *CWP1*: a gene encoding a *Saccharomyces cerevisiae* cell wall protein solubilized with *Rarobacter faecitabidus* protease I. *J Biochem* **118**: 302–311.

Shimizu J, Yoda K, Yamasaki M. 1994. The hypo-osmolarity-sensitive phenotype of the *Saccharomyces cerevisiae hpo2* mutant is due to a mutation in *PKC1*, which regulates expression of $\beta$-glucanase. *Mol Gen Genet* **242**: 641–648.

Smits GJ, Kapteyn JC, Van den Ende H, Klis FM. 1999. Cell wall dynamics in yeast. *Curr Opin Microbiol* **2**: 348–352.

Trilla JA, Duran A, Roncero C. 1999. Chs7p, a new protein involved in the control of protein export from the endoplasmic reticulum that is specifically engaged in the regulation of chitin synthesis in *Saccharomyces cerevisiae*. *J Cell Biol* **145**: 1153–1163.

Winston F, Dollard C, Ricupero-Hovasse SL. 1995. Construction of a set of convenient *Saccharomyces cerevisiae* strains that are isogenic to S288C. *Yeast* **11**: 53–55.

Zhao C, Jung US, Garrett-Engele P, Roe T, Cyert MS, Levin DE. 1998. Temperature-induced expression of yeast *FKS2* is under the dual control of protein kinase C and calcineurin. *Mol Cell Biol* **18**: 1013–1022.

Copyright © 2001 John Wiley & Sons, Ltd.
